# The Parenting Education Needs of Women Experiencing Incarceration in South Australia: Proposal for a Mixed Methods Study

**DOI:** 10.2196/18992

**Published:** 2020-08-13

**Authors:** Belinda Lovell, Mary Steen, Adrian Esterman, Angela Brown

**Affiliations:** 1 University of South Australia Adelaide Australia

**Keywords:** prison, parenting programs, education, women, mothers.

## Abstract

**Background:**

The mother-child relationship is extremely important, and for mothers experiencing incarceration, this relationship has unique challenges. There is limited evidence currently available to identify the type and content of parenting education that would best suit women who are incarcerated.

**Objective:**

This study aims to design and evaluate a parent education program for women experiencing incarceration in South Australia. The program must meet the specific needs of incarcerated women and considers the cultural needs of Aboriginal and or Torres Strait Islanders and migrant women. Hereafter Aboriginal and/or Torres Strait Islander peoples will be referred to as Aboriginal; the authors acknowledge the diversity within Aboriginal cultures.

**Methods:**

This study will utilize a mixed methods approach, including six phases framed by a community-based theoretical model. This methodology provides a collaborative approach between the researcher and the community to empower the women experiencing incarceration, allowing their parenting education needs to be addressed.

**Results:**

A scoping review was undertaken to inform this study protocol. This paper describes and discusses the protocol for this mixed methods study. Recruiting commenced in December 2019, results will be published in 2020, and the project will be completed by August 2022. This project has been supported by a Research Training Scholarship from the Australian Government.

**Conclusions:**

The scoping review highlighted a lack of rigorous evidence to determine the most appropriate parenting education program to suit women experiencing incarceration specifically, and there was little consideration for the cultural needs of women. It also became clear that when quantitative and qualitative data are utilized, the women’s voices can assist in the determination of what works, what will not work, and what can be improved. The data collected and analyzed during this study, as well as the current evidence, will assist in the development of a specific parenting education program to meet the needs of women experiencing incarceration in South Australia and will be implemented and evaluated as part of the study.

**International Registered Report Identifier (IRRID):**

PRR1-10.2196/18992

## Introduction

### Background

It is estimated that there are more than 714,000 women and girls accommodated in corrective institutions globally, comprising 6.9% of the prison population worldwide [1]. The rate of women experiencing incarceration has increased by 53% since the year 2000. The female rate of incarceration is increasing faster than the male prison population, demonstrating a 20% rise. It is also estimated that millions of children worldwide have a parent who is incarcerated, and tens of thousands live in prison with their mother [2]. Australia is following a similar trend, increasing from 2349 women in 2014 to 3494 in 2020, a 48.7% percent rise in the past 5 years [3,4]. Notably, over half of these women had dependent children under the age of 15 years in their care before incarceration [4].

Women experiencing incarceration have commonly endured complex histories that often include child abuse, sexual abuse, neglect, domestic violence, and drug and alcohol addiction [5-10], leading to a high incidence of children being removed by child protection services [5]. These life events can result in complex trauma often exhibited by low self-esteem, inability to display emotions, physical or psychological agitation, self-injury, and suicide attempts [11]. Further, this impacts the woman’s ability to maintain employment and may create issues with parenting, alcohol, and substance abuse, as well as affecting mental health [12]. These factors, combined with a lack of nurturing and inappropriate parental role modeling in childhood, can make parenting their children challenging [7]. Mothers who are incarcerated experience physical separation from their children and from their role as mothers, which impacts their identity as women [13]. Prison systems that do not provide support for mothers further damage and punish women, which can result in missed opportunities for rehabilitation, relationship building, and positive intervention [11]. Incarceration can be an important opportunity to offer women time to learn about parenting and strengthening relationships with their children [14,15].

### Aim and Objectives

This paper outlines the study protocol for the development of a parenting education program for women experiencing incarceration. The study aims to explore the parenting education needs of women experiencing incarceration in South Australia and to determine the program’s impact on the women participants. The study will address the following two questions: What are the parenting education needs of women experiencing incarceration? What is the impact of a specially designed parenting program for incarcerated women? The study will be conducted over six phases using a multiphase design, which will include a needs assessment, the development of an intervention program, and evaluation of the program.

## Methods

### Study Design

**Figure 1:**
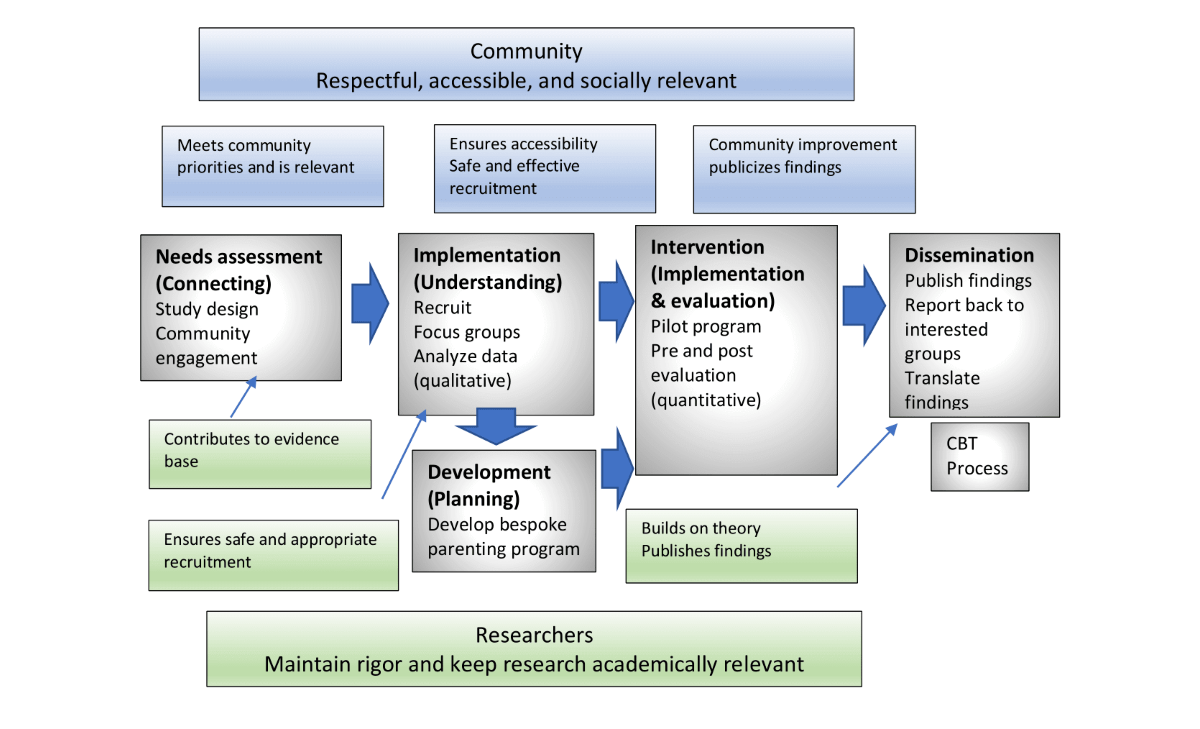
Mixed method multi-phase design framed by a community-based theoretical model (CBT). Figure adapted from Nicolaidis and Raymaker [16].

The study will utilize a mixed methods**,** multiphase design framed by a community-based theoretical model (Figure 1) [16]. This design and framework promote collaboration between the researchers, women who are incarcerated, and staff at the prison, in order to address specific problems in the target population. This methodology is an ideal fit for understanding populations that have been marginalized, giving them a voice [17,18]. A mixed methods approach using a multiphase design will involve multiple phases conducted over time with both concurrent and sequential data collection and analysis. This study will include six phases underpinned by a community-based theoretical model, involving six stages created by Stoecker [19], adapted by Badiee and Wang [17], and adapted further to suit this study by removing the medico-centric terminology.

### Phase 1

The connecting phase will involve building relationships with the prison staff to assist in developing a deeper understanding of the prison environment and build trust. Relationships will be established with Aboriginal and migrant communities to ensure the project is aligned with the principles of cultural safety considerations.

### Phase 2

The understanding phase involved the undertaking and pending publication of a scoping review, utilizing the framework outlined in the PRISMA (Preferred Reporting Items for Systematic Reviews and Meta-Analyses) extension for scoping review checklist [20]. The scoping review was undertaken to determine the outcomes of existing prison parenting education programs for women experiencing incarceration and to provide key learning outcomes for improvement of future research and development. Eleven databases were searched, and two reviewers determined the full texts that would be included. A third reviewer resolved any conflicts.

### Study Population

Incarcerated women in South Australia will be the target population of this study as well as prison employees. Participants will be eligible if they are incarcerated, able to understand and communicate English, and have a good understanding of what is involved in the study (this will be assessed when communicating with the woman). All women will have the participant information and written consent form read to them during a preparatory session, to help them understand what is involved when participating. Women who reside in two areas of the prison will be excluded from potential recruitment: women experiencing behavioral problems (8 beds) and women classified as high risk of self-harm or suicide (20 beds). Women without biological children will be included in the study, as they may also benefit from a parenting program, for example, they are currently pregnant, planning to have children in the future, or care for the children of family and friends. Staff participants will be included if they work at the prison in a role where they interact with women and are interested in being involved.

### Method of Data Collection

Focus groups will be conducted by the primary researcher and a cofacilitator (researcher). An interview schedule (with prompts) will be used by the primary researcher, to help women focus and think about what their views and needs are around parenting and parenting education. Approximately 6-8 focus groups of between 4-8 participants will be conducted with women who have varying sentences and women on remand. Additionally, three specific focus groups will be conducted for Aboriginal women (where the presence of an Aboriginal Elder provides support), pregnant women, and migrant women. The focus groups will be recorded using a digital recorder. Preliminary information and ideas will be written on flip charts by the cofacilitator, and feedback to group members will be given at the end of each session to confirm that these represent what has been discussed. Focus groups will be conducted until data saturation has been reached. After the focus groups with the women, the prison staff will be invited to attend a focus group or face-to-face interview to contribute their ideas to the program and clarify any questions that the research may have uncovered.

### Data Analysis

Descriptive thematic analysis will be used to analyze the data, which will enable a concise description of themes, allowing data organization and interpretation. Thematic analysis is a way of identifying, analyzing, organizing, describing, and reporting themes found within the data. This method of analysis provides flexibility while creating a rich, detailed, and complex interpretation of the data. This methodology will facilitate a clear and organized final report where similarities, differences, and potentially unexpected results will be presented [21]. Braun and Clarke [21] developed six stages of thematic analysis, which will be used as a guide for analyzing the data. Although this method of data analysis is a six-stage process, the analysis will involve reflection and the need to revisit stages over some time.

As guided by the Braun and Clarke [22] analytical framework, the first stage will involve transcribing the data where ideas and suggestions will be documented and cross-checked with the preliminary feedback. The second stage will involve the familiarization and immersion of data by the primary researcher and another researcher. Data that has meaning, is interesting, and contributes specifically to the development of the parenting education program will be identified and coded. The third stage will involve sorting data into themes and subthemes. During stage 4, the decision as to which themes will be combined, refined, or removed will be undertaken. This stage will ensure that data under each theme is significant, consistent, and that each theme is distinct. The next stage will involve naming the themes and creating definitions that summarise the themes. The results of the analysis will address the two research questions and create a comprehensive description of data collected, which will include direct quotes from women and staff at the study site prison.

### Phase 3

The planning phase will involve the development of a parenting education program to suit the specific needs of women experiencing incarceration in South Australia, based on needs identified by the women. An expert working group will be assembled to guide program development based on their expertise and the findings from women and staff at the prison. The University of South Australia Human Research Ethics Committee and The Department of Corrections Research and Evaluation Management Committee will review the program before implementation.

### Phase 4

A pilot program will be implemented with groups of 6-8 women in the prison, and the program will be conducted for at least one group of women.

### Phase 5

The pilot parenting education program will be evaluated using a pre-post questionnaire for the women participants to complete. This questionnaire will be designed after the program has been developed to evaluate specific aspects of the program. A combination of quantitative and qualitative data will be collected (ie, Likert scales and open-ended questions). Four to six staff members who have regular and consistent contact with the women will be interviewed after the women have participated in the program. The interviews will ascertain the program’s impact on the women, including their behavior and attitude towards parenting and the program, giving further insight into the evaluation of the program to avoid relying on self-report from the women alone.

### Phase 6

The results will be disseminated through publications and presentations. A results chapter will be written for a PhD student thesis. We envision at least four papers will be published during the undertaking of this research project, including a scoping review, the protocol, findings from focus groups, and program evaluation. Publications will be available to women and staff involved in the study and accessible via the prison library for those who are interested, and study findings and recommendations will be presented to parenting experts, female Aboriginal elders, and migrant support groups in the community.

## Results

### Phase 1

Phase 1 commenced in August 2018. The time spent building trusting relationships with the prison and the Aboriginal community was vital to this project. The effort allowed ethical approval, access to the prison, fine-tuning of research documentation, recruiting assistance, providing a space for interviews, allowing digital recording, connecting with key staff members, and engaging with key members of the Aboriginal community to ensure the project continues to follow guidelines for respectful research [23].

### Ethical Considerations

This project was approved by the University of South Australia Human Research Ethics Committee on August 29, 2019, with approval until August 29, 2022 (application ID: 201956). The Department of Corrections Research and Evaluation Management Committee in South Australia also approved the project from November 12, 2019, to November 12, 2020. Ethics approval was gained by the Central Adelaide Health Local Health Network Human Research Ethics Committee from the 25th of June for one year. The approval includes the connecting, understanding, and planning phases of the research.

Prison staff will not be involved in recruiting women in order to avoid recruitment bias. The researchers will recruit women following an information session. This process will reduce power imbalance relationships with prison staff who may influence women’s decision to participate. Written consent for all aspects of the project will be obtained. The vulnerability of incarcerated women has been taken into careful consideration in all phases of this research project. Women will be provided with an information session, as described earlier, where a detailed description of the study will be provided, ensuring that participation is voluntary and allowing the women to make an informed choice regarding whether they would like to be involved. Particular care will be taken to ensure confidentiality of data collected and participants’ anonymity. Pseudonyms will be used, and limited demographics will be collected to ensure that the women cannot be identified. The researchers will wear a duress alarm during the focus groups and will have the ability to contact security for immediate response. If the women become distressed, the researchers will refer the women to the appropriate supports, guided by a referral flow chart. The staff participants will be recruited via an email introduction from an administrator to reduce recruitment bias, which will include the participant information sheet and consent form to read. They will also be invited to an information session during which the researcher will explain the study, and the women can decide if they would like to attend a focus group. Deidentified electronic data will be stored on a password-protected computer, and written transcripts and demographic data will be stored in a locked cabinet in a locked office at the University of South Australia (C4-45). Documents will be stored for 7 years after the publication of the results, per the General Disposal Schedule No. 24, Universities of SA [24]. Digital recordings will be deleted after transcription.

### Phase 2

The scoping review, unlike previous reviews, focused on the impact of parenting education for incarcerated women as well as highlighting the qualitative data and outlining the content of the programs to determine what can be learned for future program design. During this phase, challenges with the prison system, program content, what worked and what did not, was identified. The review identified limited rigorous research evaluating parenting education programs for women in prisons; currently, it is unknown what the most appropriate content of a parenting program for incarcerated women should entail. Limited consideration has been given to the specific cultural needs of incarcerated women and how these needs can be met. There are also very few programs developed after first identifying the needs of women. The challenges experienced by other researchers has been noted as a learning strategy for working in prisons. Cultural safety was not taken into consideration in the majority of studies. In studies that did adapt a program to suit the cultural needs of Aboriginal women, it was not specifically evaluated to determine if the needs of Aboriginal women were met. There were few programs where the input of women was sought in the development phase of a program, which would appear to be an ideal place to start designing a program for a population with unique needs. Therefore, in this study, a ground-up approach will be utilized, where women are questioned about their parenting education needs, and a parenting education program will be developed and designed around their specific circumstances and identified needs. The scoping review is expected to be published in 2020.

Focus groups will be conducted in the prison from December 2019 to December 2020, and the results of these interviews will be published in 2020.

### Phases 3-5

These phases are reliant on the completion of the preceding phases.

### Phase 6

Dissemination of the results will occur throughout the project. The project will be completed by August 2022. This project is funded by an Australian Government Research Training Scholarship for 3 years beginning in August 2018.

## Discussion

### Overview

Findings demonstrate a limited number of parenting programs for incarcerated women that have been developed with input from women in the target population. Many of the women who find themselves involved with the criminal justice system have experienced difficult childhoods themselves. It is, therefore, challenging for many of the women to parent their own children positively. The women may not have had the opportunity to access parenting support education in the community, and the challenges of separation can create further difficulties with the mother-child bond and relationship. Imprisonment is an opportunity for some women to change their lives and access learning opportunities that are offered in prison. One of the most important elements to improve outcomes for women is to initiate and maintain relationships with family and children [25,26]. Despite the many challenges that women encounter, children are a strong motivator to avoid reoffense and substance abuse and the desire to regain custody [27].

The possible benefits of this study may result in women feeling positive and empowered about contributing their ideas and suggestions to a parenting education program, which may help women experiencing incarceration in the future. Some women involved in the preliminary phases may have the opportunity to attend the pilot parenting education program, which may have a positive impact on women’s knowledge about parenting and, by extension, positively impact the next generation of children.

### Conclusion

Parenting education is important as the separation between mothers and their children can have serious emotional, physical, and psychological effects on both the mother and child. Effective parenting programs have the potential to assist in the promotion of healthy relationships and reduce the intergenerational cycle of poor parenting and incarceration. This information must be disseminated to improve the education provided to women experiencing incarceration and their children. The data collected from participating women and staff in prison will be utilized in a ground-up approach to design a specific parenting education program to meet the needs of incarcerated women.
